# Supplementary material for: A Bayesian approach to modeling phytoplankton population dynamics from size distribution time series
Source: PLoS Comput Biol. 2022 Jan 14;18(1):e1009733. doi: 10.1371/journal.pcbi.1009733 (PMC8794270; doi:10.1371/journal.pcbi.1009733)
Supplement: S1 Text — Describes stability of model estimates across 48-hour sliding windows. (PDF) [file pcbi.1009733.s001.pdf]

S1 Sliding window experiments

As part of our observation sensitivity experiments, we modified the start time of the model fitting to examine the resulting changes in parameter estimates. As the original cell size distribution dataset only contains two days of data, we appended the dataset to itself to create a 96-hour time series. This allowed us to fit models to a sequence of two-day continuous cell size distribution data that start at different times of the laboratory-simulated light-dark cycle (Fig S1 A. The start times of these windows ranged from 2 to 46 hours and were spaced four hours apart. In each experiment, the model initialization time is set to match the start time of the window and data outside the window is discarded.

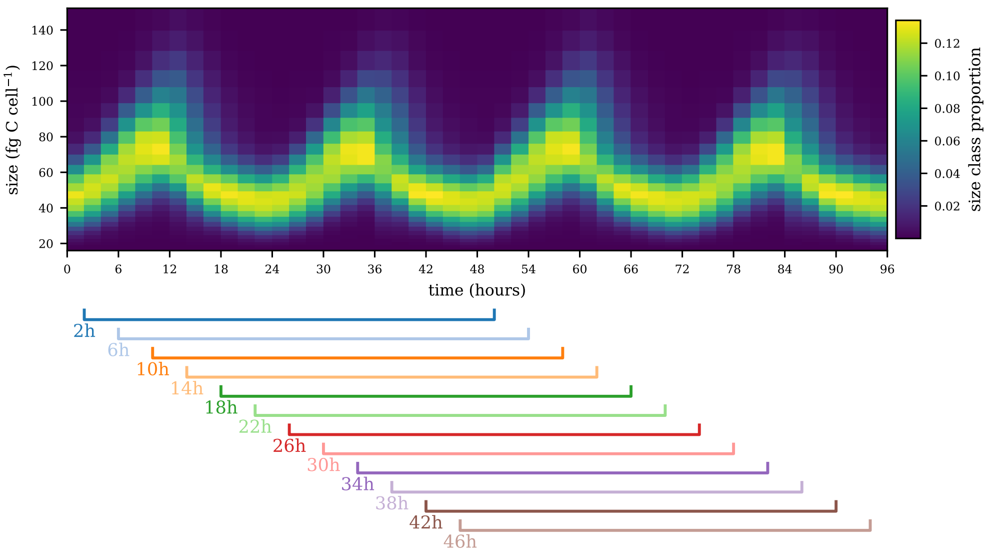

Fig S1 A. The extended size distribution dataset used in the sliding window experiments and the 2-day windows in which the models are fit.

Here we focus on results for model m_{bmb}, which are representative for most of our models; when individual models deviate from these results, we note it in the text. Results for all models can be found in the accompanying GitHub repository [1]. Overall, parameter estimates remain consistent for most start times, but we noted a weak cyclical pattern in estimated values and an outlier estimate for a start time of 26 hours (Fig S1 B), which are both examined below.

The pattern is aligned with the daily cycle and is characterized by increased division and decreased carbon loss rates at start times near 10 hours and – 24 hours later – near 34 hours (Fig S1 B). It is driven by the estimation of initial conditions at a start time with a large model-observation misfit, which is aligned with the peak of the cell size distribution in most of our models. At the peak of the cell size distribution, for example at t=34 h, the m_{bmb} daily cycle underestimates the *Prochlorococcus* cell size distribution (Fig S1 E). When the estimation window starts at a peak, the estimated initial conditions deviate strongly from the daily cycle steady state solution (compare the solutions of the 2 hours and 10 hours start time at t=10 h, or the 10 hours start time solution at t=10 h and t=34 h; Fig S1 ED). Due to the increase in the initial cell

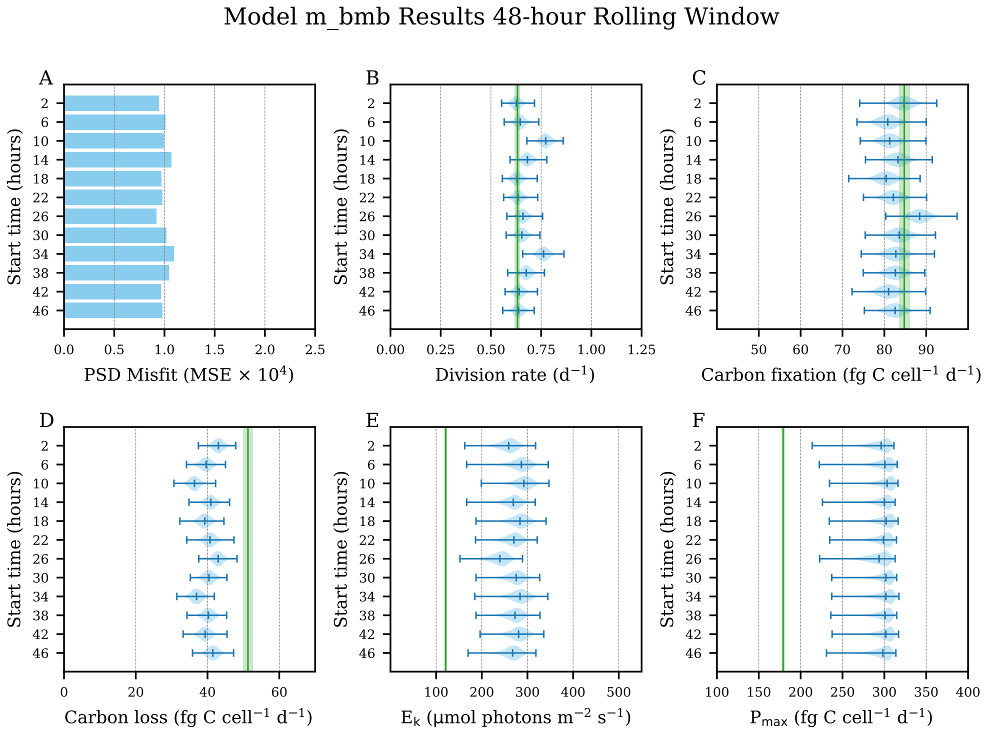

Fig S1 B. Rate parameter estimates of the model m_{bmb} for each window of the sliding window experiment.

size distribution, the division rate, which increases with cell size, becomes inflated, impacting other parameter estimates accordingly. This effect is more pronounced for models with a larger model-data discrepancy, while models that fit the size distribution better throughout the daily cycle, such as m_{ftf}, show a weaker cyclical pattern in the parameter estimates (Fig S1 C).

Some models, such as m_{bmx} and m_{btb} (Fig S1 D), showed much more volatility in their parameter estimates among windows. This indicates that these models may be more unstable, and hence their results may be less reliable than the other models.

The second noteworthy pattern in the m_{bmb} estimates is the parameter estimate for the start time of 26 hours. Here, the model fitting procedure converged to solutions with higher average carbon fixation and higher carbon loss compared to simulations at other start times. A likely cause for this pattern is the strong correlation structure between the model parameters (Fig 6 in the main document) combined with the broad priors in our model specification. As a result, changes in the start time and associated changes in the order of the observations, in combination with different initial conditions, can lead to changes in the posterior estimates that may appear as outliers with respect to the other sliding window experiments. We observed this type of outlier infrequently for most models, but it occurred more often for m_{btb} which also showed worse convergence properties in our other experiments.

To summarize the stability of our models, we plotted the daily division rate for each model in each window against the concentration parameter σ (Fig S1 F). The vertical spread of each cluster corresponds to the variability of the daily division rate, whereas the horizontal spread corresponds to the variability of the concentration parameter. In general, models with greater values of σ exhibited less variability in their daily division rates across windows.

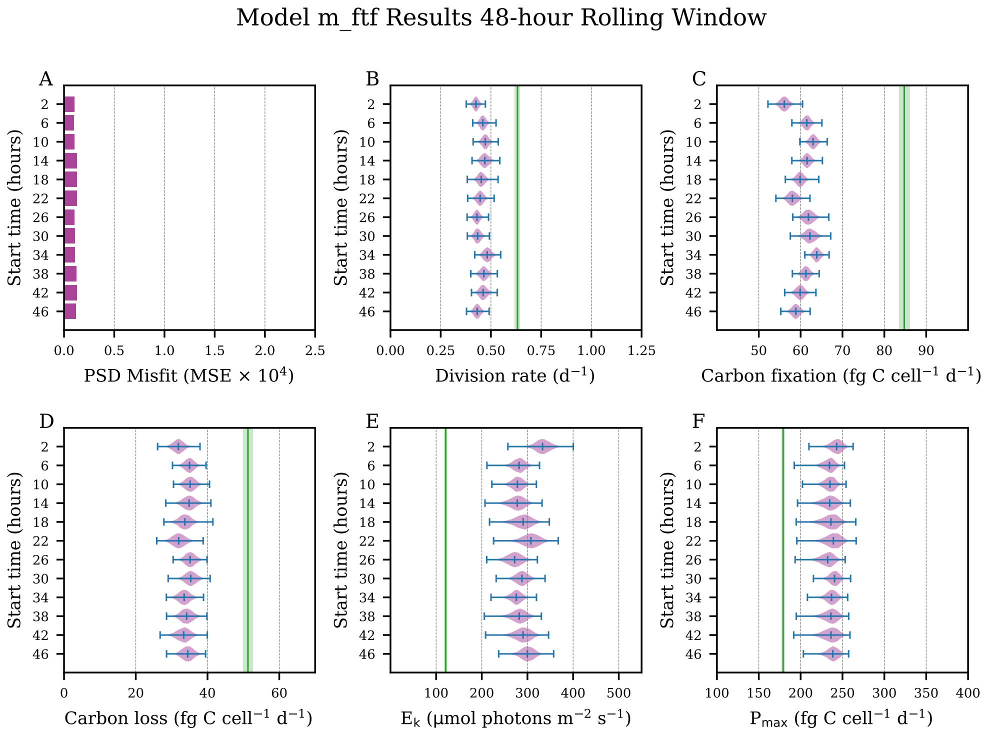

Fig S1 C. Daily rate parameter estimates of the model m_{ftf} for each window of the sliding window experiment. This model showed greater stability in its parameter estimates across windows compared to simpler models such as m_{bmb}.

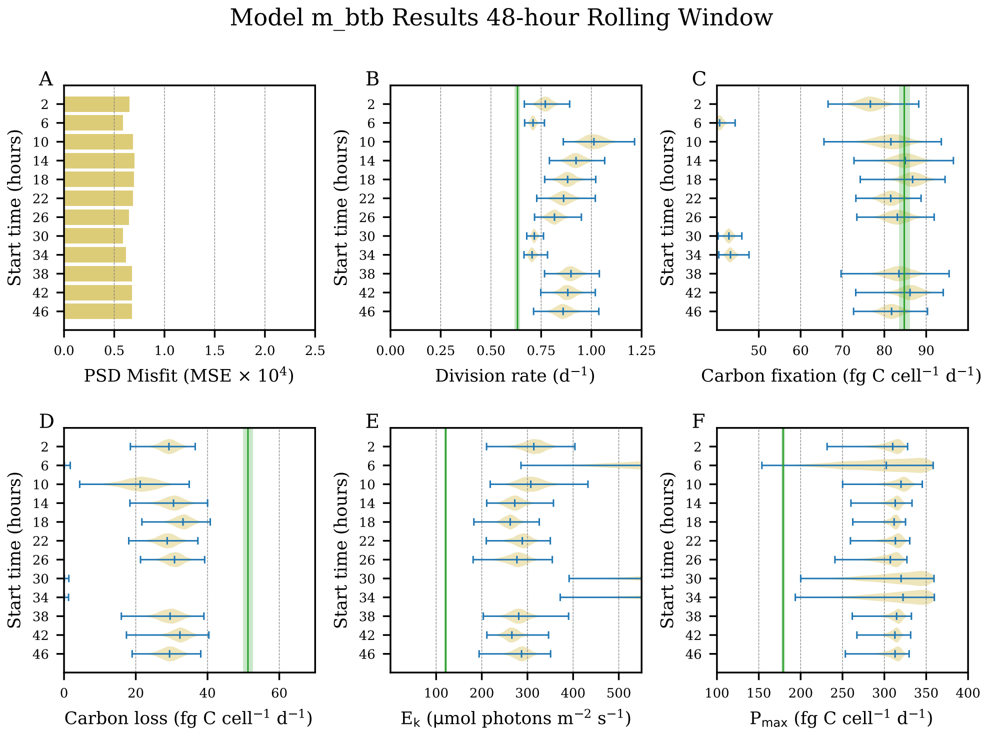

Fig S1 D. Daily rate parameter estimates of the model m_{btb} for each window of the sliding window experiment. Model results were much more volatile for this model and m_{bmx} than the others.

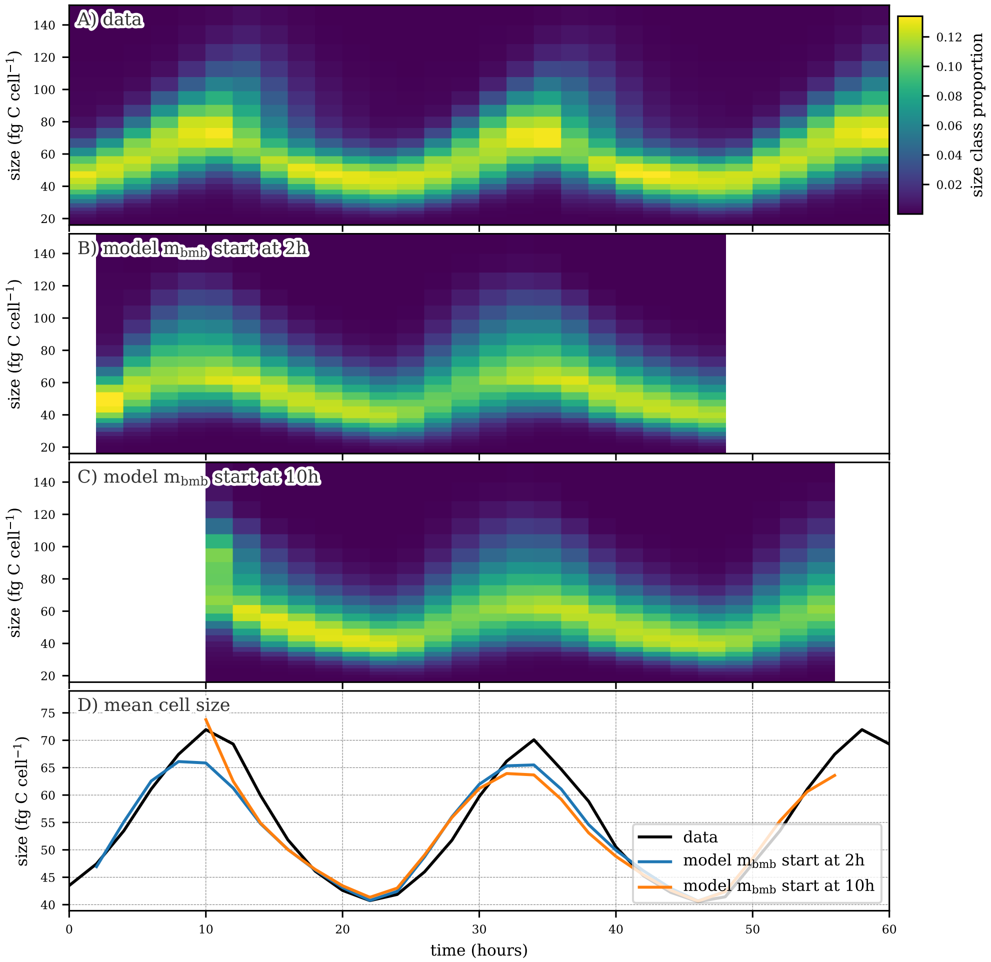

Fig S1 E. Size distribution in the (A) data, the model m_{bmb} in the rolling window experiment started at (B) hour 2 and (C) hour 10. (D) The evolution of the mean cell size in data and model.

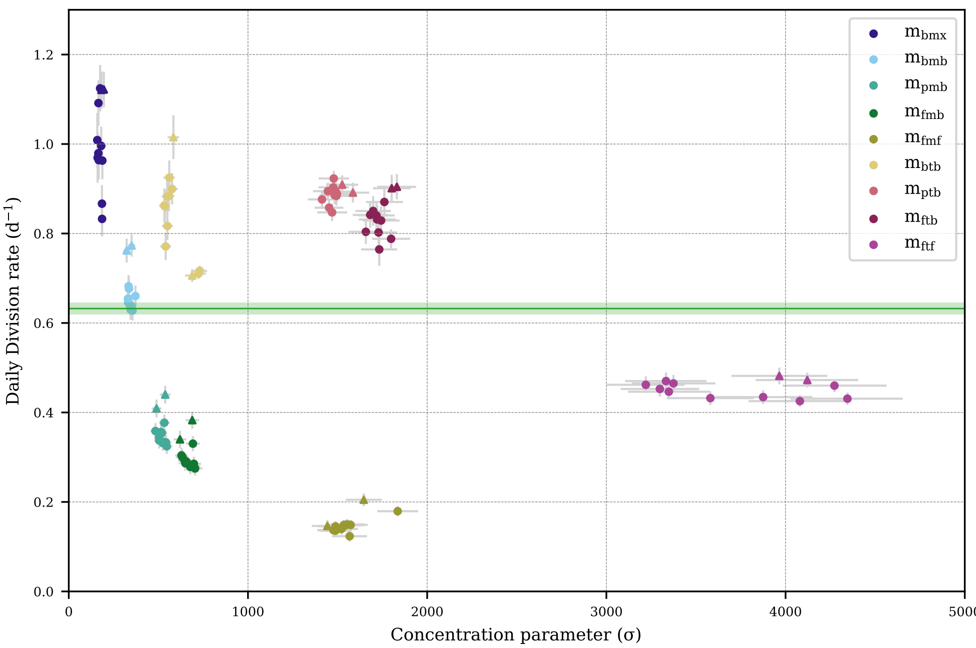

Fig S1 F. Sliding window experiment daily division rates vs. concentration parameter σ by model. Gray error bars indicate one standard deviation of the posterior distribution. Each data point represents the posterior mean daily division rate from one window. Windows starting at $t = 10$ and $t = 34$ are represented as triangles. Green horizontal line indicates observed daily division rate. Green shaded area indicates one standard deviation of uncertainty around the observed value.

References

1. GitHub repository with data, material and results for “A Bayesian approach to modeling phytoplankton population dynamics from size distribution time series”. GitHub:`https://github.com/CBIOMES/bayesian-matrix-population-model`.
