# Supplementary material for: A Bayesian approach to modeling phytoplankton population dynamics from size distribution time series
Source: PLoS Comput Biol. 2022 Jan 14;18(1):e1009733. doi: 10.1371/journal.pcbi.1009733 (PMC8794270; doi:10.1371/journal.pcbi.1009733)
Supplement: S2 Text — Describes model results when removing data points from the training set and examines model performance in predicting the held-out data. (PDF) [file pcbi.1009733.s002.pdf]

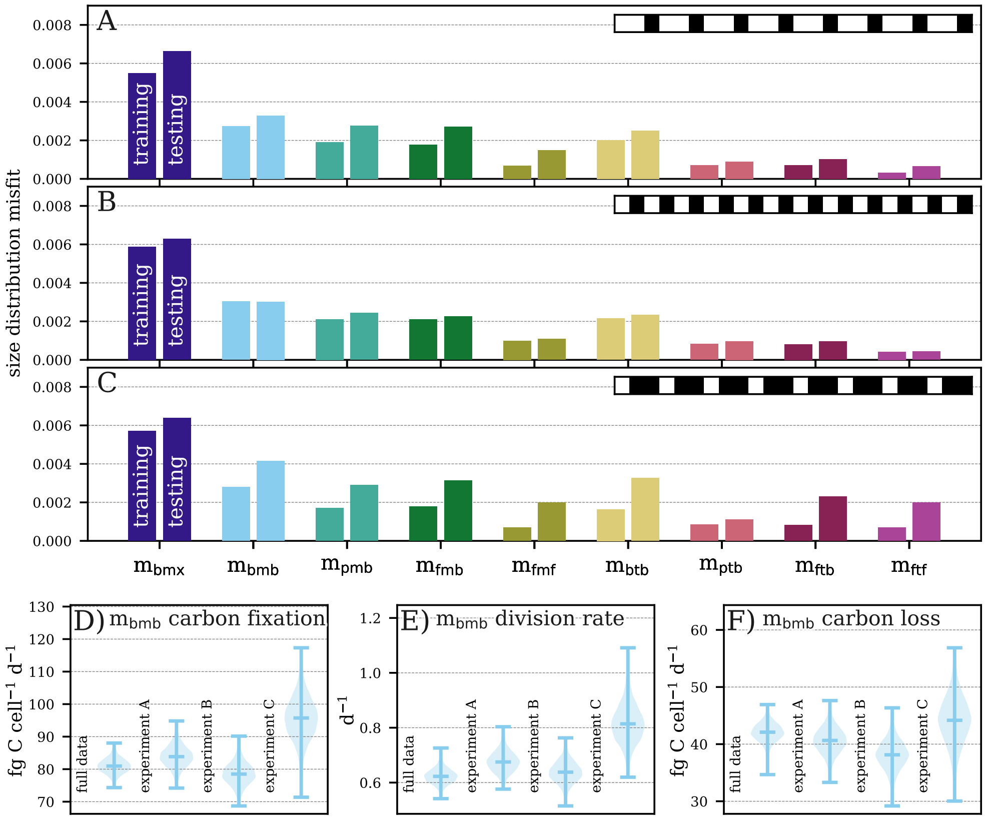

Fig S2 A. Holdout validation experiments. Size distribution misfit for testing and training data (left and right bar) for each model in the cross-validation experiments (A), (B), and (C) with top right corner visualizing the indices of the testing (black) and training data (white). Examples of the posterior distributions of select model parameters for the full dataset and the two cross-validation experiments: (D) daily carbon fixation rate for m_{bmb}, (E) daily division rate for m_{bmb}, and (F) daily carbon loss rate for m_{bmb}.

S2 Hold-out validation

In experiment A, the data from every third time step were removed, in experiment B data were removed from every other time step, and in experiment C, two-thirds of the data were removed (see top right corner of Fig S2 A A, B, C). As expected, the error on the training data reflected model complexity and decreased from m_{bmx} to m_{fmf}, and again for the models with time-dependent division m_{btb} to m_{ftf}, in all three experiments (Fig S2 A A,B,C). While the ratio of testing to training data error increased for more complex models, the absolute value of the testing data error did not increase with model complexity in most of our experiments. The exception involved m_{ptb} and m_{ftb}, which differ only in their size-dependent growth parameterizations. While the more complex m_{ftb} with the free growth parameterization exhibited a lower training data error, m_{ptb} model with power-law growth achieved a lower testing data error. Taken

together with the results for m_{pmb}, which were similar to those of m_{fmb}, we have some evidence that the power-law growth parameterization is suitable for models in this application, creating a size-dependent growth relationship that performed better on testing data than a freely estimated relationship.

Reducing the number of observations in the training set had a noticeable impact on the models parameter estimates (Fig S2 A D-F). With less data in the training dataset, the posterior distributions of the estimated parameters broadened from those obtained using the full dataset and eventually showed shifts in the mean parameter estimates when more data is excluded (e.g. m_{bmb} daily division in experiment C, Fig S2 A E). The broadening matches our intuition: fewer observations constrain the parameter estimates to a lesser extent than the information contained in the full dataset. With two thirds of the data excluded and observations occurring every 6 hours, the rate parameters could no longer be estimated reliably and mean parameter estimates deviated noticeably from their values on the full dataset. In summary, when as much as one half of the data was removed, the estimated rate parameters still capture the daily cycle of *Prochlorococcus* dynamics. Estimates for the parameters of interest also remained stable.

Table S2 A. All models.

**Model***	**Growth**	**Division**	**Loss**
m_{bmx}	**b**asic	**m**onotonic	**x** (no loss)
m_{bmb}	**b**asic	**m**onotonic	**b**asic
m_{pmb}	**p**ower-law size-dependence	**m**onotonic	**b**asic
m_{fmb}	**f**ree size-dependence	**m**onotonic	**b**asic
m_{fmf}	**f**ree size-dependence	**m**onotonic	**f**ree size-dependence
m_{btb}	**b**asic	**t**ime-dependent	**b**asic
m_{ptb}	**p**ower-law size-dependence	**t**ime-dependent	**b**asic
m_{ftb}	**f**ree size-dependence	**t**ime-dependent	**b**asic
m_{ftf}	**f**ree size-dependence	**t**ime-dependent	**f**ree size-dependence

*The letters in the subscript of the model name denote the growth, division, and loss parameterizations used in the model, respectively.

S2 Daily rate estimates

Here, we examine the daily rate of all nine models we tested (Table S2 A). Again, the MSE of the estimated cell size distribution decreased as the number of model parameters increased (Fig S2 BA), though this did not correlate with better daily rate estimates. Of the four models not shown in the main text, the time-dependent division models (m_{btb}, m_{ptb}, m_{ftb}) overestimated the daily division rate (Fig S2 BB), while m_{fmb} underestimated this quantity. Model m_{btb} accurately captures daily carbon fixation (Fig S2 BC), though the instability observed in this model suggests that it is not reliable in general. The other models not shown in the main text (m_{fmb}, m_{ptb}, m_{ftb}) underestimated carbon fixation. While m_{fmb} was able to accurately estimate carbon loss, the time-dependent models underestimate this quantity; in fact, m_{ptb} and m_{ftb} estimated essentially no carbon loss (Fig S2 BD). Model m_{fmb} also accurately estimated E_k and $P_{\max}$, while the time-dependent models tended to overestimate these quantities, with the exception of m_{ptb}, which underestimated $P_{\max}$ (Fig S2 BE, F).

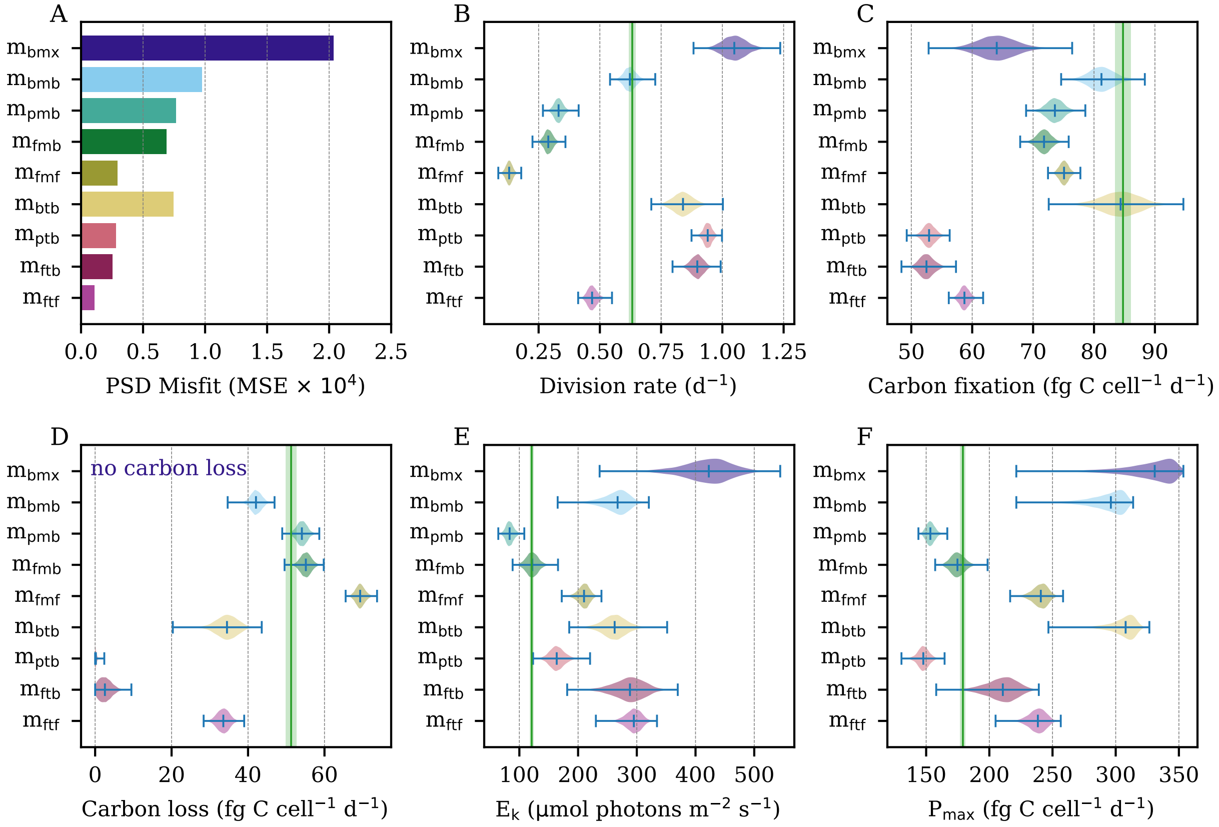

Fig S2 B. Model estimated daily rate parameters. (A) Mean squared error (MSE) of estimated proportions to the observed particle size distribution (PSD). (B) Estimated daily division rates. (C) Estimated daily carbon fixation. (D) Estimated daily carbon loss. (E) Estimated photosynthetic saturation parameter. (F) Estimated maximum photosynthetic rate. (B-F) Green vertical lines indicate ground truth calculated from data. Green shaded areas indicate uncertainty surrounding ground truth measurements. Model estimates shown as posterior distributions.
