# Supplementary material for: A Bayesian approach to modeling phytoplankton population dynamics from size distribution time series
Source: PLoS Comput Biol. 2022 Jan 14;18(1):e1009733. doi: 10.1371/journal.pcbi.1009733 (PMC8794270; doi:10.1371/journal.pcbi.1009733)
Supplement: S3 Text — Results of generating synthetic data from mbmb and mftf and fitting the corresponding model to recover the underlying parameters. (PDF) [file pcbi.1009733.s003.pdf]

S3 Estimating parameters from synthetic (model-generated) data

The accuracy of model estimated rate parameters was assessed by conducting a series of model fitting experiments using model-generated synthetic data. That is, we run a model with known parameters, extract the simulated size distribution from the model output, add some noise, and use the resulting synthetic data for model fitting. The model's ability to accurately estimate the known parameter values used to generate it provides a best-case estimate of the model's ability to recover parameters from "real" data (all dependent on how the experiment is set up, the amount of noise added to the synthetic data, the time-frequency at which the data is extracted, etc.). This type of experiment can also highlight parameter-dependent differences in the model's estimation ability, which can aid interpretation and assessment of model results.

We performed a series of 7 synthetic data experiments for m_{bmb} and m_{ftf}. Observations were generated at the same time resolution as the lab measurements (see Fig 3A), noise was added using the same Dirichlet-Multinomial statistical model introduced in Observation model (setting $\sigma = 10\,000$; the synthetic data is shown in [1]). The results from these experiments (Fig S3 A and S3 B) can be summarized in 4 main points: (1) Parameters without strong correlations to other parameters, such as ρ_{max}, can be identified accurately. (2) Parameters with strong correlations, such as γ_{max} and E_k, are more difficult to recover; similar difficulties were encountered with $\mathrm{P}_{\mathrm{max}}$and E_k on the laboratory dataset, see Fig 6). (3) While some of the underlying parameters, such as δ_{max}, may be difficult to identify, we can still obtain accurate estimates of the associated rate parameter, i.e daily division rate, daily carbon fixation, and loss rate parameters. (4) The spread of the posterior samples (for example quantified by its standard deviation) is a good indicator for the accuracy of the parameter estimate. That is, if a given parameter cannot be identified well by the sample mean, the sample spread is typically large. These results suggest that our MPMs are able to accurately recover model-generated daily rate estimates using only light and size distribution data. However, since we tested our models only a single dataset, the accuracy of the daily rate estimates may change in other, non-model-generated datasets.

References

1. GitHub repository with data, material and results for "A Bayesian approach to modeling phytoplankton population dynamics from size distribution time series". GitHub:`https://github.com/CBIOMES/bayesian-matrix-population-model`.

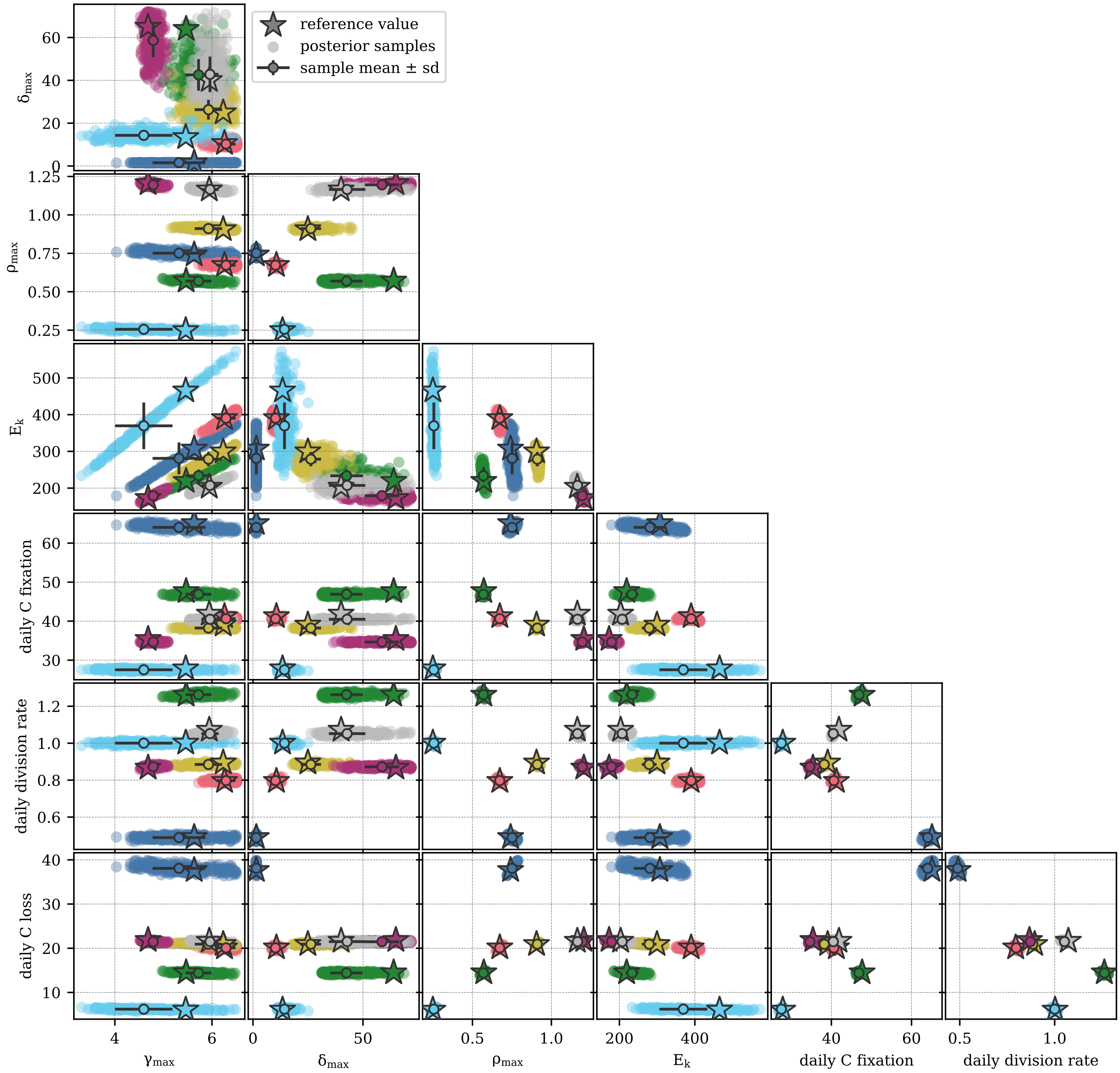

Fig S3 A. Parameter estimation from synthetic data for $m_{\mathbf{bmb}}$. Scatter plots of the posterior samples, the sample means and standard deviations, and the reference values (used to generate the synthetic data) for select parameters for the model m_{bmb}. Colors denote different estimation experiments, each with distinct reference values.

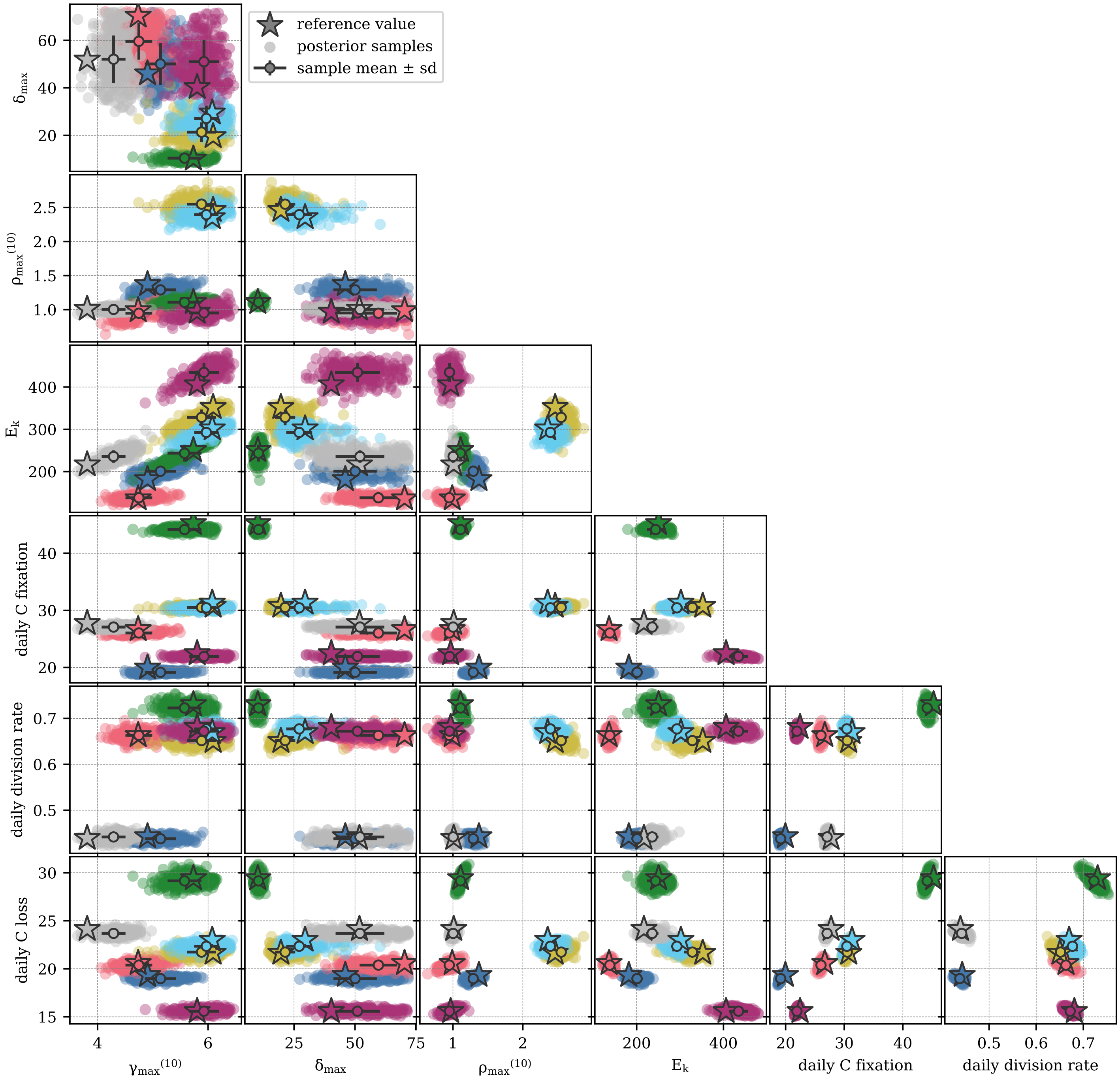

Fig S3 B. Parameter estimation from synthetic data for $m_{\mathbf{ftf}}$. Scatter plots of the posterior samples, the sample means and standard deviations, and the reference values (used to generate the synthetic data) for select parameters for the model m_{ftf}. Colors denote different estimation experiments, each with distinct reference values. For m_{ftf}, some parameters have size-dependence, and $\gamma_{\text{max}}^{(10)}$ and $\rho_{\text{max}}^{(10)}$ denote their 10th value.
