# Supplementary material for: A Bayesian approach to modeling phytoplankton population dynamics from size distribution time series
Source: PLoS Comput Biol. 2022 Jan 14;18(1):e1009733. doi: 10.1371/journal.pcbi.1009733 (PMC8794270; doi:10.1371/journal.pcbi.1009733)
Supplement: S1 Table — (PDF) [file pcbi.1009733.s004.pdf]

Table S1. All models.

Model*	Growth	Division	Loss
m_{bmx}	**b**asic	**m**onotonic	**x** (no loss)
m_{bmb}	**b**asic	**m**onotonic	**b**asic
m_{pmb}	**p**ower-law size-dependence	**m**onotonic	**b**asic
m_{fmb}	**f**ree size-dependence	**m**onotonic	**b**asic
m_{fmf}	**f**ree size-dependence	**m**onotonic	**f**ree size-dependence
m_{btb}	**b**asic	**t**ime-dependent	**b**asic
m_{ptb}	**p**ower-law size-dependence	**t**ime-dependent	**b**asic
m_{ftb}	**f**ree size-dependence	**t**ime-dependent	**b**asic
m_{ftf}	**f**ree size-dependence	**t**ime-dependent	**f**ree size-dependence

*The letters in the subscript of the model name denote the growth, division, and loss parameterizations used in the model, respectively.
