# Supplementary figures and images for: A Bayesian approach to modeling phytoplankton population dynamics from size distribution time series
Source: PLoS Comput Biol. 2022 Jan 14;18(1):e1009733. doi: 10.1371/journal.pcbi.1009733 (PMC8794270; doi:10.1371/journal.pcbi.1009733)

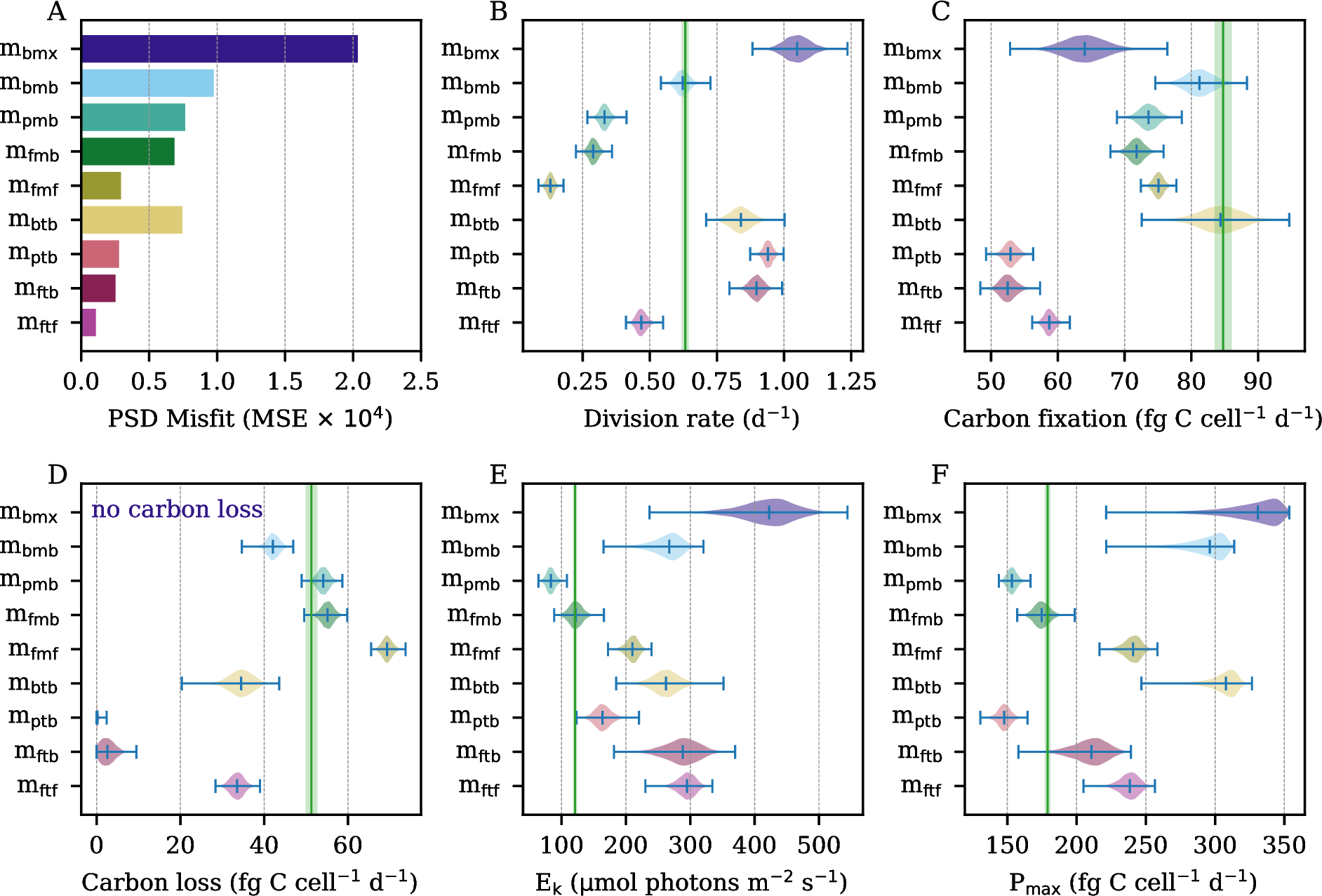

Supplement: S1 Fig — (A) Mean squared error (MSE) of estimated proportions to the observed particle size distribution (PSD). (B) Estimated daily division rates. (C) Estimated daily carbon fixation. (D) Estimated daily carbon loss. (E) Estimated photosynthetic saturation parameter. (F) Estimated maximum photosynthetic rate. (B-F) Green vertical lines indicate ground truth calculated from data. Green shaded areas indicate uncertainty surrounding ground truth measurements. Model estimates shown as posterior distributions. (TIF) [file pcbi.1009733.s005.tif]

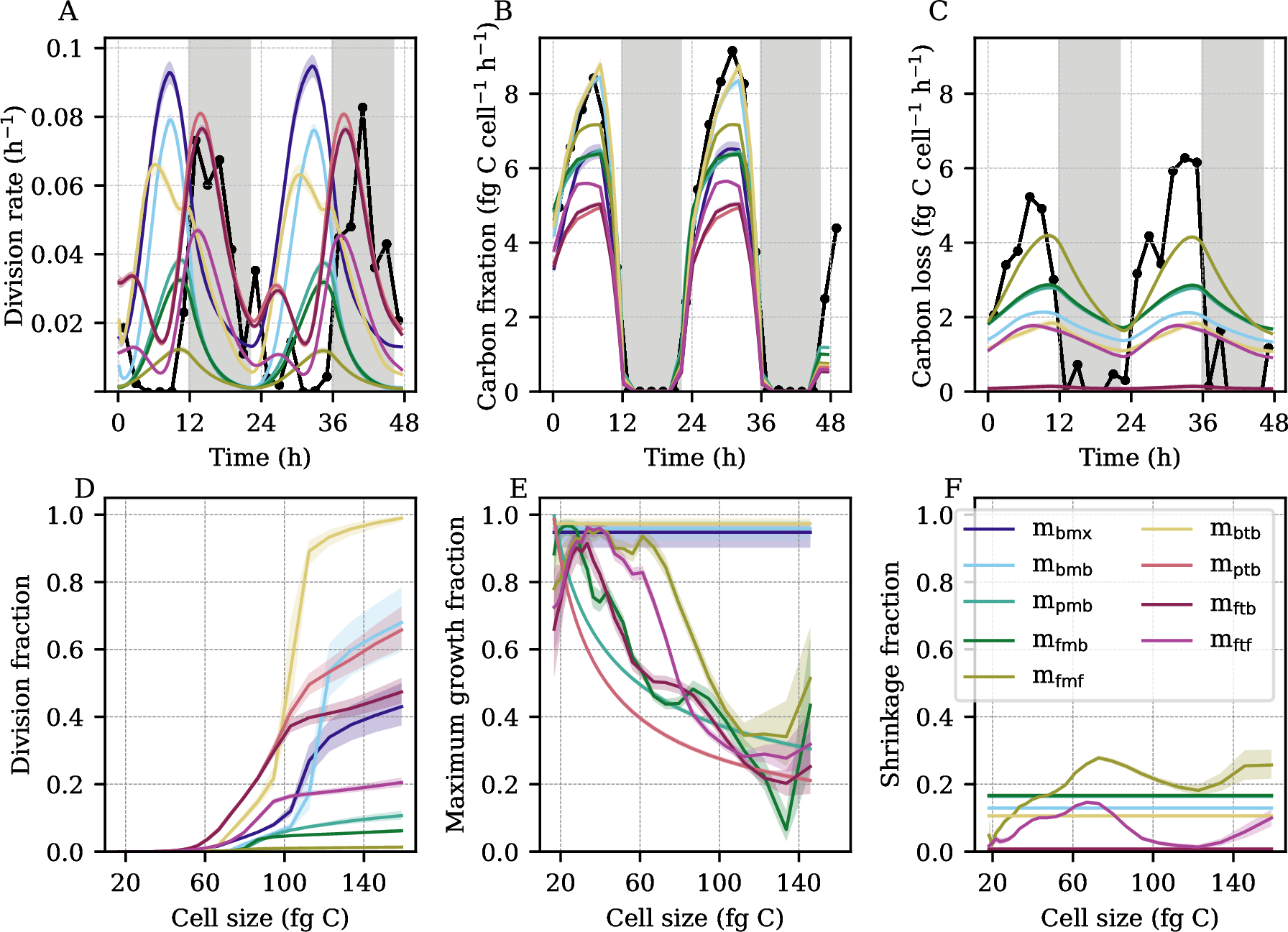

Supplement: S2 Fig — (A) Observed (black) and estimated (colored bands) hourly division rates. (B) Observed (black) and estimated (colored bands) hourly carbon fixation. (C) Observed (black) and estimated (colored bands) hourly carbon loss. (A-C) Black points indicate ground truth calculated from data. (D) Estimated cell division fraction as a function of cell size. (E) Estimated light-saturated cell growth (carbon fixation) fraction as a function of cell size. (F) Estimated cell shrinkage (carbon loss) fraction as a function of cell size. (A-F) Colored bands indicate model estimates. Shading indicates the first to third quartiles of the posterior distributions. (D-F) Fractions correspond to MPM transitions over a 20-minute time period. (TIF) [file pcbi.1009733.s006.tif]

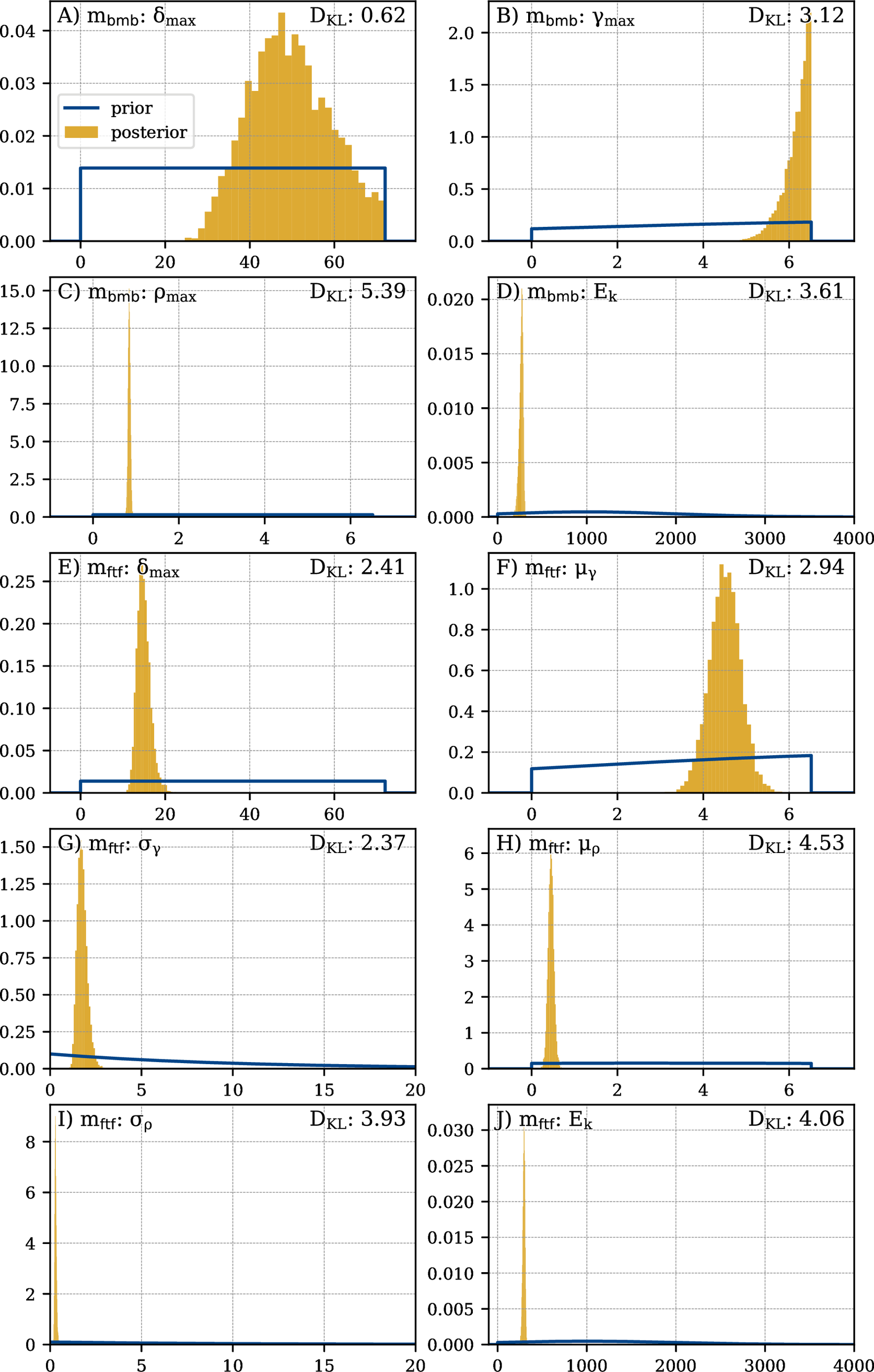

Supplement: S3 Fig — Prior pdf and histogram of posterior samples for select biological parameters of models mbmb (A-D) and mftf (E-J). The Kullback–Leibler divergence of the marginal distribution (DKL) quantifies the difference between prior and posterior distribution for each parameter. (TIF) [file pcbi.1009733.s007.tif]

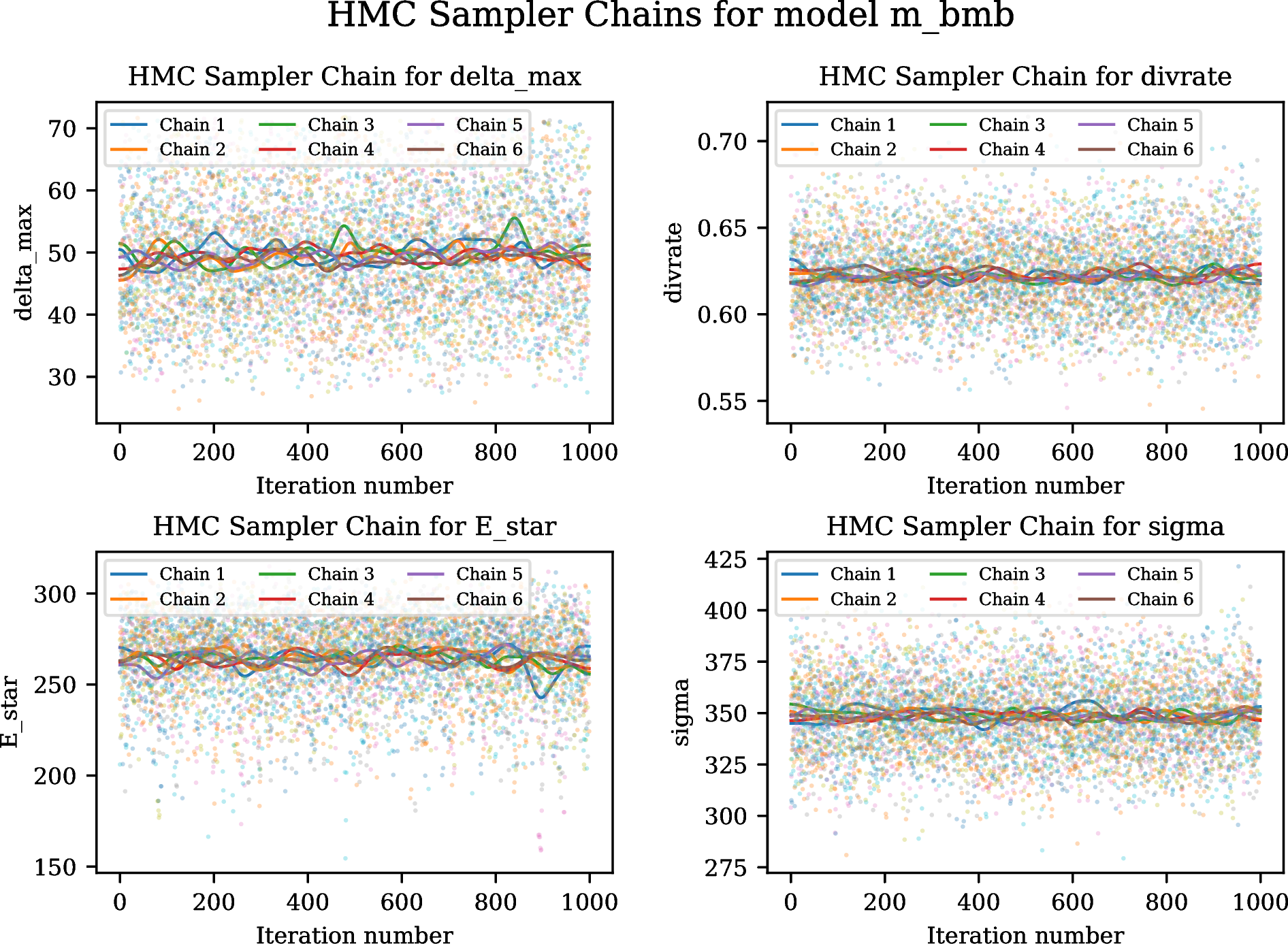

Supplement: S4 Fig — Each of six parallel post-warmup sampling chains for four parameters of mbmb. Points indicate individual samples and solid lines represent Gaussian smoothers. Each color corresponds to one of the six chains. (TIF) [file pcbi.1009733.s008.tif]

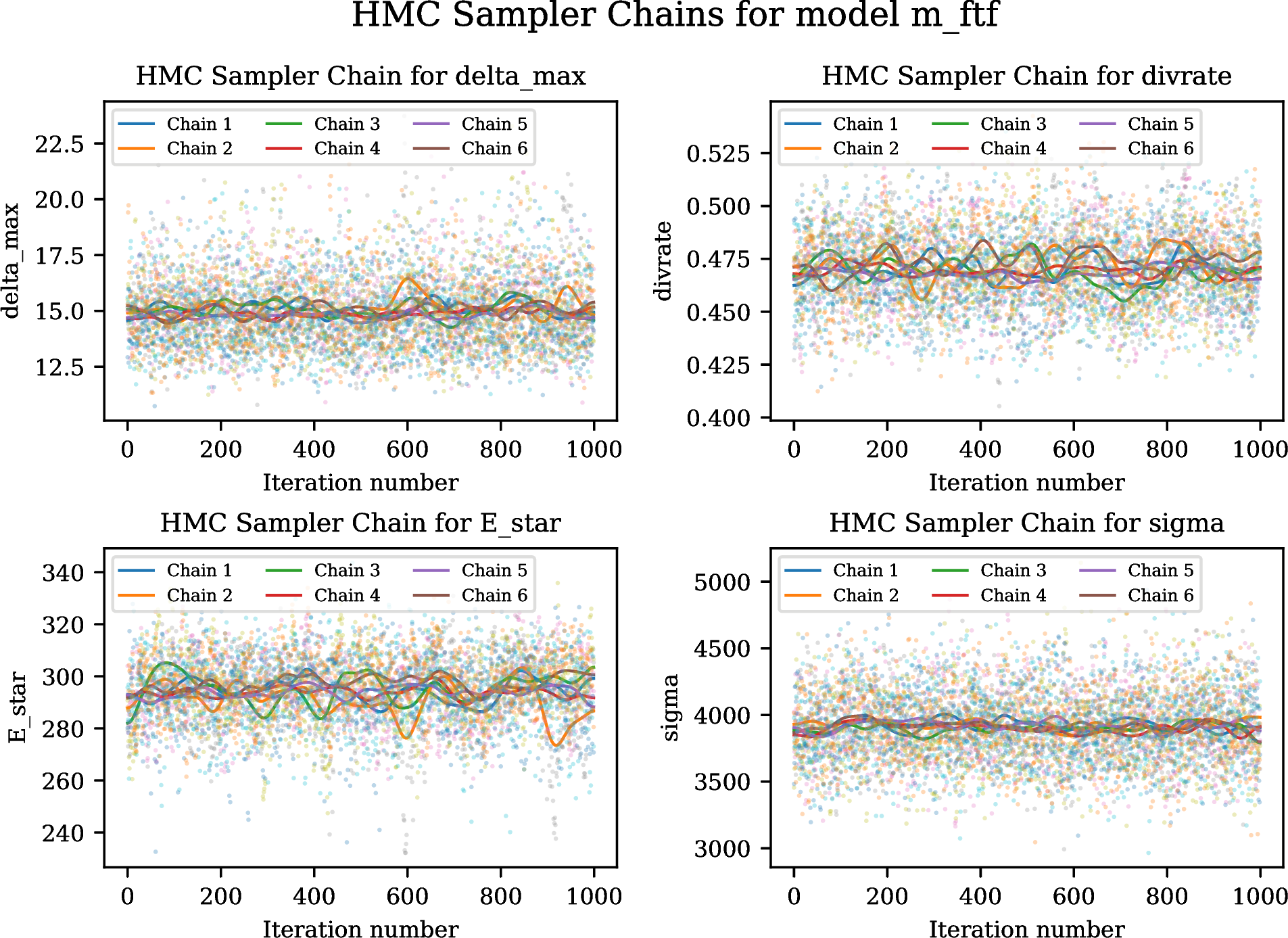

Supplement: S5 Fig — Each of six parallel post-warmup sampling chains for four parameters of mftf. Points indicate individual samples and solid lines represent Gaussian smoothers. Each color corresponds to one of the six chains. (TIF) [file pcbi.1009733.s009.tif]
