# Supplementary material for: A Bayesian approach to modeling phytoplankton population dynamics from size distribution time series
Source: PLoS Comput Biol. 2022 Jan 14;18(1):e1009733. doi: 10.1371/journal.pcbi.1009733 (PMC8794270; doi:10.1371/journal.pcbi.1009733)
Supplement: Attachment — Submitted filename: response_to_reviewers.pdf [file pcbi.1009733.s010.pdf]

Comments of reviewer 1 and responses

Major comments

(M1.1) comment: This paper explores and investigates several different formulations of a size-structured matrix population model to accurately estimate growth rate from hourly cell size distributions. Different model formulations are checked against laboratory data of the marine cyanobacterium Prochlorococcus to infer how well each model version estimates growth rate and other physiological relevant parameters.

The expansion of model formulations presented here is a valuable and worthwhile effort; if it is possible to capture additional physiological parameters of a phytoplankton population from cell-size distributions, the data would be quite informative. The authors attempt to expand and improve upon earlier models whose underlying formulas for transition matrices were not able to be linked directly to the cellular parameters of interest here (carbon fixation, carbon loss, and cell division).

However, identifiability issues raises serious concerns about whether model formulations and Bayesian approach are able to obtain valid inference:

A Bayesian approach requires that the evidence, $P(\{n\})$, is constant in order to use the proportionality listed after equation 6, line 418. How can you ensure this partition function is constant?

We thank the reviewer for the comments. By definition, $P(\{n\})$, the evidence, is an average with respect to the parameters of interest $\boldsymbol{\theta}, \sigma$, and it is therefore constant with respect to these parameters, see [42]. Here, we are not integrating over different model parametrizations, which may differ in $\boldsymbol{\theta}$, but performing the inference for each model individually. In response, we have added the following statement to the revised manuscript:

revised text (Section "Observation model", par. 1): [...] the proportionality holds because the evidence $p\left(\{\boldsymbol{n}_k\}_{k=0}^{K-1}\right) = \int\int_{\boldsymbol{\theta},\sigma} p\left(\{\boldsymbol{n}_k\}_{k=0}^{K-1} | \boldsymbol{\theta}, \sigma\right) \pi(\boldsymbol{\theta}, \sigma) d\boldsymbol{\theta} d\sigma$ is constant with respect to the model parameters $(\boldsymbol{\theta}, \sigma)$ [42].

(M1.2) comment: Instability was encountered when attempting variational inference, which may indicate that the procedure is not producing valid estimates of the posterior using the likelihood and prior models. Authors do not provide posterior distributions for all parameters (only two are shown in Figure 6) nor how these estimates correspond to assumed priors, (e.g. for parameters E_k and $\rho_{\max}$ given in Table 2); the presentation is not clear on whether posteriors are reasonable with respect to expert-choice priors. The presentation can be better if the reader is more directly convinced that the likelihood with chosen priors do in fact result in STAN sampling a realistic (rather than just an algebraically emergent) posterior. Perhaps the authors could include both the prior and posterior distributions (on the same plot) of a sensitive or difficult to constrain parameter from Table 2.

We encountered instability when using Stan for variational inference; however, the Hamiltonian Monte Carlo (HMC) technique we employed for all experiments presented in the manuscript did not exhibit instability. The HMC procedure did converge for all of our models, which we verified using the $\hat{R}$ convergence diagnostic, where we ensured that $\hat{R} < 1.05$ for all models, as noted in Implementation. We only used variational inference to offer a scalable alternative to HMC. We mentioned instabilities encountered in variational inference in the manuscript to provide full transparency to our inference procedure and justify our use of HMC. In response, we reworded the sentence in question to clarify that the instabilities did not affect the results we present in the manuscript. In addition, as suggestion by the reviewer, we included a new figure showing the prior and posterior distributions for key biological parameters of models m_{bmb} and m_{ftf} in the Supporting Information (S3 Fig) to provide further evidence that HMC produced valid estimates.

... To this end, please show:

- Maximum likelihood estimates of parameters to show that priors and posterior estimates are reasonable; MLE should give similar values to mean posterior.

We thank the reviewer for this suggestion. We have now added the following discussion regarding likelihood inference for models m_{bmb} and m_{ftf} in the Implementation section:

revised text (Section "Implementation", par. 3): In order to benchmark our results, we used Stan's optimization to compute the maximum likelihood estimator (MLE) for m_{bmb} and m_{ftf}. However, the results were unstable and sensitive to initialization. To investigate the sensitivity of our inference to the prior distributions, we implemented m_{bmb} and m_{ftf} with flat priors, so that the posterior distribution is proportional to the likelihood. For m_{bmb}, this gave virtually identical results. For m_{ftf}, the model failed to converge, indicating that stronger prior information is necessary to remove potential identifiability issues introduced by the additional parameters for size- and time-dependent processes.

... - MC sampler chains (i.e. as Reference 38, Figure 4) for the most difficult to constrain parameter to affirm 'burn-in', feasible models, and successful exploration of posterior.

We have now included sampler chain results for four model parameters of models m_{bmb} and m_{ftf}, see Fig **??** and Fig **??**, respectively, that show that each chain appears to have reached its stationary distribution and that the chains are well-mixed, as suggested by the $\hat{R}$ values.

... - Plots of posterior probability for all parameters.

We have now included S3 Fig, showing the prior and posterior distributions for key biological parameters of models m_{bmb} and m_{ftf}.

(M1.3) comment: Please more fully discuss risk of over-parameterization. The paper itself ends with the admission that size-distribution data cannot constrain all the parameters. It is entirely possible that no model is able to accurately partition cell size changes between division, cell growth and carbon loss, so it is unclear how the authors are accounting for this and refining their model structures. It is also unclear why so many models are presented when some are very likely over-parameterized, especially the free-size dependence versions. The paper would benefit from a streamlining and selection of models, with over-parameterized models noted in the SI. Please also discuss benefits and risks of incorporating more concrete cell biology within the model. For example, a time delay on cell division or no carbon loss during night would likely provide more simpler model formulations.

We thank the reviewer for this suggestion, which we think greatly improved the quality of the manuscript. We have reduced the number of models in the main text from nine to five, with four others noted in the Supporting Information (S1 Table, S1 Fig, S2 Fig). Model m_{bmx} was included as it most closely represents past work. Model m_{bmb} was included as it adds respiration and performs best on daily rates. We included m_{pmb} because of evidence in the literature suggesting a power-law relationship between cell size and carbon fixation [29]. Model m_{fmf} was included to allow for free size dependence in all carbon flux rates and m_{ftf} was added to demonstrate the effect of adding time-dependent division, as motivated by our discussion of division vs. cell size. Finally, we have added a discussion of the trade-offs between our simplest models and the most complex models in our work :

revised text (Section "Discussion", par. 5): Ultimately, the choice of model will depend on the goal of the particular application. Our simpler models offered greater interpretability and accuracy of daily rate parameters, while more complex models were able to recover the timing of cell division at the cost of additional computation time and the requirement of stronger prior information.

Specific comments

(S1.1) comment: Title: The title as written is misleading; 'flexible', as written, addresses the Bayesian approach utilized, which appears to be standard. The title also suggests that the paper is about inference techniques for size-structure matrix population models (a very large class of models!), which is not the case. The flexibility that the authors are mentioning is presumably for model construction (not the inference procedure). The models implemented here are also highly specific to high-resolution, time series flow cytometry data of phytoplankton. The title should reflect this; please revise.

We agree with the reviewer and have modified the title accordingly. The new title is: "A Bayesian approach to modeling phytoplankton population dynamics from size distribution time series".

(S1.2) comment: Line 23-24: Awkward/misplaced sentence; in-situ growth rate estimates are not really obscured by heterotrophic biomass nor detritus; it is because these rates cannot be obtained from abundance or carbon estimates alone (which are a composite of growth and loss). Please clarify.

We rephrased the sentence accordingly, it now reads:

revised text (Section "Introduction", par. 1): Direct *in-situ* measurement of this quantity cannot be obtained from abundance or carbon biomass alone, which are a composite of cell growth, cell mortality, and other biological and physical processes.

(S1.3) comment: Line 58-61: Authors are incorrect with regards to how previous models were evaluated. While goodness of fit is checked, model formulations in some papers were benchmarked against laboratory division rate data. Please revise. A more objective presentation of earlier studies is also needed across the manuscript; authors of previous model papers are presumably well-aware of their model limitations, and physiological measurements weren't often the goal in these past studies. Risk of over-parameterization is only one of many reasons why other versions were not explored. Please do not assume intent unless explicitly stated in these earlier manuscripts.

It was not our intention to prescribe intent to the authors of previous work, though we understand how this may have come across in the text. We agree with the reviewer that a more thorough interpretation of past studies would improve the quality of the manuscript. To this end, we have made modifications throughout the manuscript (e.g. in the abstract, author summary, and introduction) to discuss in more detail the context of previous applications and our efforts to build upon these studies.

(S1.4) comment: Line 91: Ending sentence here is not entirely correct; there is no connection to the larger marine carbon cycle in this paper. The rate parameters perhaps may offer this, but the authors do not extrapolate to any larger cycles in this manuscript.

We agree, and have removed the reference to the marine carbon cycle and rephrased the passage in question:

revised text (Section "Introduction", par. 5): Finally, we converted model parameters to estimates of biological rates such as carbon fixation and carbon loss, allowing for more direct interpretation of estimated parameter values.

(S1.5) comment: Lines 100-104: I'm not sure I entirely follow the arguments and the emphasis placed on cell size in relationship to hourly division rate. It is correct to compare plots of hourly division rate to mean cell size? Division produces an increase of small cells, such that a plot between mean cell size and division rate would not necessarily show a correlation (no large cells are expected with higher division rates, and after division, which must have happened to get a rate, cell size is no longer correlated to the process).

We wanted to directly show the relationship between cell size and division rate in the data to help the reader understand why size-structured MPMs impose a size-dependence on the division rate and to motivate a time-dependent division parameterization. We now mention the observed relationship with the mean extends to the 70th, 80th, 90th, and 95th percentiles, where a strong correlation to the hourly division rate at a 6-hour lag can still be observed. We have incorporated this new information in the manuscript text and modified Fig 3C. The updated figure panel now shows two cell size distributions that have a similar tail, i.e. similar proportions of large cells, rather than a similar mean. Despite the similar proportion of large cells, which in the model would lead to similar division rates, the measured division rates differ markedly.

... Furthermore, as the authors have count data in each size class, could the authors not present an analysis of how cell sizes shift in comparison to hourly division rate? For example, do the largest cells decrease in abundance immediately after dusk (as suggested by Fig.2), whereas medium cells decrease in abundance more towards the middle of the night? And is this all accompanied by corresponding increases in smaller cells? This would probably yield better insight into the timing of division and guidance for model division formulas. I agree that the process of cell division is not likely instantaneous and complete cell fission will likely take a few hours.

We agree with the reviewer that an analysis of how cell sizes shift in comparison to division rates would be very interesting. However, we feel that this is beyond the point we want to communicate to the readers, namely that cell division (i.e. cytokinesis) is not instantaneous, and therefore cell size and cell division are not tightly coupled on hourly time scales (if they were, the highest division rates would occur at the peak of the population cell size). We feel that the new information we added to Fig 3 now better communicates this point to the readers.

(S1.6) comment: Discussion: The paper examines performance of each model version, but at the end, the reader is left wondering what perhaps is the best formulation going forward or where exactly the work should be going. Could the authors add additional recommendations or concrete decisions to guide readers?

We thank the reviewer for the suggestion. We have now added a discussion regarding the potential application of the models to future datasets:

revised text (Section "Discussion", par. 5): Ultimately, the choice of model will depend on the goal of the particular application. Our simpler models offered greater interpretability and accuracy of daily rate parameters, while more complex models were able to recover the timing of cell division at the cost of additional computation time and the requirement of stronger prior information.

revised text (Section "Discussion", par. 5): Here, we tested our models on a highly synchronized population of *Prochlorococcus* grown under laboratory conditions, but we expect natural populations to be less synchronized and exhibit noisier dynamics over the diel cycle. Additional processes not accounted for in this study, such as grazing and viral lysis, which could potentially affect phytoplankton size distributions, will need to be tested. The application of our models to field data will be addressed in future work.

(S1.7) comment: Methods: Please provide example functional curves for cell growth, cell division and cell loss; these are useful for reader visualization and how different parameters affect each function.

We thank the reviewer for this suggestion. In response, we have added to the manuscript a new figure (Fig 7) that illustrates the relevant functional curves and parameters that govern the models' size-, light-, and time-dependence.

(S1.8) comment: Methods: Perhaps I missed it, but explicit code to call Stan with model formulations does not appear available.

We had included a reference to the repository only at the end of the manuscript; we have now added another reference to the Implementation section in the Materials and methods.

Comments of reviewer 2 and responses

Major comments

(M2.1) comment: The work presented in this manuscript represents an important advance in the use of demographic models for understanding the ecology of marine phytoplankton. It should eventually be published. There are, however, a few issues that need to be addressed in the current version of the manuscript.

One contribution of this paper is the application of Bayesian methods for parameter estimation to a size-structured matrix population model for marine phytoplankton. The application of Bayesian methods for matrix models is not particularly new. A second is the extension of the model to allow for the shrinking of cells (as a result of respiration). This second part is new. I would, the introduction of shrinking into the model introduces new parameters. I would like to see some evidence that these parameters can be estimated accurately from simulated data (where the true values are known). My intuition is that there are many combinations of the parameters that would produce the same sequence of size distribution, and, as a result identification may be an issue. The authors touch on this in the section that begins on line 258.

We thank the reviewer for recommending the addition of simulated (synthetic) data to the manuscript, which provides valuable insights into the parameter estimation results. In response, we have conducted simulation studies for m_{bmb} and m_{ftf} in which we simulate data from these models and fit the generated data; the results highlight that identifiability issues can occur when parameters are strongly correlated, yet that the two models we tested could accurately estimate the most important daily rate parameters. We have added a reference to the results in the manuscript and provided details in the Supporting Information (S3 Text).

(M2.2) comment: There is no free lunch: one must pay for the Bayesian approach by the specification of prior distributions on the parameters. In my opinion the authors do not discuss this cost sufficiently in the discussion. What does one do when there is no "prior knowledge?" How sensitive are the posterior distributions to the priors? This second question is important, and could (should?) be addressed with the simulation studies, but should also be addressed in estimation of the parameters for the laboratory data.

We agree with the reviewer that the selection of prior distributions deserves more emphasis in the manuscript. In response, we have added a discussion of prior selection in the manuscript:

revised text (Section "Discussion", par. 2): The selection of priors is a requirement for the Bayesian inference procedure. When scientific knowledge is available to determine plausible parameter values, this can be formally incorporated into the inference and resulting estimates; otherwise, uninformative priors [43] can be used, which include broad uniform distributions or the so-called Jeffreys prior [44]. However, constraining complex models with uninformative priors may lead to poor identifiability and numerical instability. In this case, it may be useful to conduct additional studies to learn about plausible parameter ranges so that information can be brought into model fitting or use an approach known as Empirical Bayes, which aims to construct a prior distribution that is consistent with the data before formally fitting the model [45].

We have also added more details on prior sensitivity for models m_{bmb} and m_{ftf} in the Implementation section:

revised text (Section "Implementation", par. 3): To investigate the sensitivity of our inference to the prior distributions, we implemented m_{bmb} and m_{ftf} with flat priors, so that the posterior distribution is proportional to the likelihood. For m_{bmb}, this gave virtually identical results. For m_{ftf}, the model failed to converge, indicating that stronger prior information is necessary to remove potential identifiability issues introduced by the additional parameters for size- and time-dependent processes.

Finally, we have reordered the sections in Materials and methods and added text to clarify which quantities are model parameters (and thus have associated prior distributions) and which quantities are functions of these parameters.

(M2.3) comment: In a number of places in the manuscript (eg., lines 58-61, 346-348) where the authors claim that previous work was flawed because that previous work used a measure of goodness of fit to observed size distributions "as a proxy for overall model performance". At least for references [18], [19], [23] and [24] this was not the case. Instead, they judged model performance by how well the model could estimate division rate – the object of inference – compared with a "gold standard" method (dilution experiments) by calculating concordance.

We agree with the reviewer that a more thorough discussion of the context of past work would improve the quality of the manuscript. To this end, we have made modifications throughout the manuscript (e.g. in the abstract, author summary, and introduction) to discuss in greater detail the context of previous applications and our efforts to build upon these studies.

Note: We have changed the format of the Supporting Information to meet the requirements of the journal. Each item of Supporting Information has now been uploaded as a separate file. A description of each item can be found at the end of the manuscript, after the references.
